# A Simple Separation Method of the Protein and Polystyrene Bead-Labeled Protein for Enhancing the Performance of Fluorescent Sensor

**DOI:** 10.1155/2018/8461380

**Published:** 2018-07-11

**Authors:** Hye Jin Kim, Dong-Hoon Kang, Seung-Hoon Yang, Eunji Lee, Taewon Ha, Byung Chul Lee, Youngbaek Kim, Kyo Seon Hwang, Hyun-Joon Shin, Jinsik Kim

**Affiliations:** ^1^Department of Clinical Pharmacology, Kyung Hee University, Seoul 02447, Republic of Korea; ^2^Center for Bionics, Korea Institute of Science and Technology, Seoul 02792, Republic of Korea; ^3^Systems Biotechnology Research Center, Korea Institute of Science and Technology, Gangneung 25451, Republic of Korea; ^4^Department of Medical Biotechnology, Dongguk University, Seoul 04620, Republic of Korea; ^5^Center for Nano-Photonics Convergence Technology, Korea Institute of Industrial Technology (KITECH), Gwangju 61012, Republic of Korea; ^6^Center for BioMicrosystems, Korea Institute of Science and Technology (KIST), Seoul 02792, Republic of Korea

## Abstract

Dielectrophoresis- (DEP-) based separation method between a protein, amyloid beta 42, and polystyrene (PS) beads in different microholes was demonstrated for enhancement of performance for bead-based fluorescent sensor. An intensity of ∇|*E*|^2^ was relative to a diameter of a microhole, and the diameters of two microholes for separation between the protein and PS beads were simulated to 3 *μ*m and 15 *μ*m, respectively. The microholes were fabricated by microelectromechanical systems (MEMS). The separation between the protein and the PS beads was demonstrated by comparing the average intensity of fluorescence (AIF) by each molecule. Relative AIF was measured in various applying voltage and time conditions, and the conditions for allocating the PS beads into 15 *μ*m hole were optimized at 80 mV and 15 min, respectively. In the optimized condition, the relative AIF was observed approximately 4.908 ± 0.299. Finally, in 3 *μ*m and 15 *μ*m hole, the AIFs were approximately 3.143 and −1.346 by 2 nm of protein and about −2.515 and 4.211 by 30 nm of the PS beads, respectively. The results showed that 2 nm of the protein and 30 nm of PS beads were separated by DEP force in each microhole effectively, and that our method is applicable as a new method to verify an efficiency of the labeling for bead-based fluorescent sensor ∇|*E*|^2^.

## 1. Introduction

Labeling is one of the essential processes for analyzing and tracking the biomolecules and proceeds by conjugating the molecules with various materials such as isotope markers [1], photochromic compounds [2], and fluorescence polystyrene (PS) beads [3–5]. Especially, fluorescent PS beads have competitive price, high accessibility, and controllability so that various biomolecules such as protein [6], cell [3], and deoxyribonucleic acid (DNA) [7] have been conjugated with fluorescent PS beads followed by being quantified and qualified [8–10]. Qin et al. separated the protein which conjugated with surface-modified fluorescent PS [11], and Fakih et al. multidetected the viral DNA using gold nanoparticle-coated fluorescent PS beads [12]. However, fluorescent PS beads are not conjugated with biomolecules perfectly; in other words, their labeling efficiency is under 100%, and consequently, not only biomolecules conjugated with fluorescent PS beads but also nonconjugated biomolecules existed in the analyte. The nonconjugated biomolecules decrease the accuracy and sensitivity in analyzing and tracking of the biomolecules; hence, a method is required for separating the biomolecules conjugated with PS beads and nonconjugated biomolecules, namely, the residue molecules. So, residue biomolecules after labeling need to be separated from the biomolecules, which are conjugated with labels, ideally. Although centrifugation approaches [13, 14] and fluidic-based approaches [15] are suitable for separating the residue molecules, these approaches are complex and require an additional process.

Dielectrophoresis (DEP), resulting from inhomogeneous electric fields, has been utilized for the specific manipulation of the particles, cells, and viruses as well as biomolecules such as DNA and even single protein, because of its simplicity, efficiency, and usability [16–18]. The intensity of the DEP force is dominated by the size of the molecules and the strength of the electric fields, which occurred between the electrodes, so that various molecules are affected by a different intensity of the force according to the size of the molecules and the structure of the electrode. Lapizco-Encinas et al. concentrated and separated the live and dead bacteria with insulator-based DEP (iDEP) [19]. But two types of bacteria were separated according to different types of DEP force, negative DEP and positive DEP, and Chen et al. suggested a simplified dielectrophoretic-based microfluid device for particle separation [20]. But these approaches were limited for observing the various molecules simultaneously or consisted of the complex structure.

Here, we suggest a simple method to separate the nonconjugated protein, namely, the residue protein, and the protein conjugated with PS beads with the DEP force, which is applicable for verifying the efficiency of labeling between protein and PS beads. The protein and PS beads were separated into two microholes with different diameters and formed on a single electrode according to the intensity of DEP force induced. The intensity of the DEP force increased when the diameter of the microhole was smaller, and thus, stronger repulsive force and attractive force occurred in the small microhole than the large one. Consequently, the smaller molecules, residue protein, were allocated into the small microhole, whereas the bigger molecules, PS beads, were expelled from that small microhole followed by allocating into the large microhole; it means that the protein and PS beads were separated. To verify the separation between protein and PS beads, approximately 2 nm of the protein, amyloid beta 42, and 30 nm fluorescent PS beads were used. The diameter of the two microholes for separating the protein and PS beads was optimized to 3 *μ*m and 15 *μ*m, respectively, by calculating the intensity of ∇|*E*|^2^ in each microhole with COMSOL simulation. Also, an applied voltage to induce the DEP force was optimized to 80 mV, which induced a difference in the ∇|*E*|^2^ force approximately 9.059-fold between two microholes. The microholes were fabricated by microelectromechanical systems (MEMS) technique, and separation between the protein and polystyrene beads was demonstrated by comparing a relative averaged intensity of fluorescence (AIF) by each protein and each PS bead.

## 2. Materials and Methods

### 2.1. Theory

The molecule present in an inhomogeneous electric field, *E*, is influenced by the DEP force, *F*_DEP_, which is expressed as follows [21]:

(1) $$\begin{array}{c} F_{\text{DEP}}=2\pi r^{3}\varepsilon_{m}K\left(\omega \right)\cdot \nabla {\left|\vec{E}\right|}^{2}, \end{array}$$

where *r*, *ε*_m_, and *K*(*ω*) represent the radius of molecules, the effective permittivity of liquid, and the Clausius–Mossotti factor, respectively. The *E* and gradient of the electric field ∇|*E*|^2^ are described as follows:

(2) $$\begin{array}{c} \vec{E}=-\frac{\Delta V}{\Delta \vec{d}}, \end{array}$$

(3) $$\begin{array}{c} \nabla \left|\vec{E}\right|=\frac{\partial^{2}V}{\partial d_{x}^{2}}\hat{x}+\frac{\partial^{2}V}{\partial d_{y}^{2}}\hat{y}+\frac{\partial^{2}V}{\partial d_{z}^{2}}\hat{z}, \end{array}$$

where *V* and *d* are the applying voltage and the distance between the electrodes, respectively. On the basis of (1) and (3), the intensity of the DEP force can be modified through (4) as follows:

(4) $$\begin{array}{c} F_{\text{DEP}}\propto \frac{r^{3}}{d^{2}}. \end{array}$$

The intensity of ∇|*E*|^2^ is calculated with a finite-element model (FEM) in the AC/DC module of COMSOL Multiphysics software 5.2 (COMSOL Inc., USA).

### 2.2. Materials

Thirty nanometres of the carboxylate-modified polystyrene (PS) bead labeled with fluorescence (Sigma-Aldrich Inc., Korea) and 2 nm of the TAMRA-labeled beta-amyloid (1–42) protein (AnaSpec Inc., USA) were used to verify the separation of molecules, whose excitation/emission wavelength (*λ*_ex_/*λ*_em_) was ∼470/505 nm and ∼544/572 nm, respectively. The protein and PS beads were diluted with 1 mM PBS buffer (Corning Korea Co. Ltd., Korea) to create a 1 ng·mL^−1^ solution.

### 2.3. Fabrication of Microholes

Microholes were fabricated by a standard MEMS process. First, an insulation layer, 300 nm of SiO_2_, and an electrode layer, 30 nm of tantalum (Ta) and 150 nm of platinum (Pt), were sequentially deposited on the 4-inch silicon (Si) wafer by thermal oxidation and sputtering, respectively. Next, an AZ GXR 601 photoresist (AZ Electronic Materials, Luxembourg) was coated by a spin coater (30 s, 3000 rpm) and exposed (3.8 s, 12 mW·cm^−2^). Then, the hole patterns were etched by inductively coupled plasma etching (Oxford Instruments), and the photoresist was stripped by Microwave Plasma Asher (Plasma-Finish, Germany).

### 2.4. System Setup for Molecules Separation and Fluorescence Analysis

A DG4062 Series waveform generator (Rigol Technologies Inc., USA) (frequency range: up to 60 MHz; voltage range: up to 10 V), which applies a sinusoidal AC voltage for inducing the DEP force in the microhole on a single electrode, was used. The intensity of fluorescence was observed via an electron-multiplying charge-coupled device (ANDORiXonEM), an oil immersion 100x lens (Nikon Corp., Japan) (NA: 1.4), and an Eclipse Ti inverted microscope (Nikon Corp.) equipped with a halogen lamp and a 593 nm (bandwidth: 40 nm) filter and was analyzed by Image-Pro Plus 6.0 (Media Cybernetics Inc., USA). The average intensity of fluorescence (AIF) was calculated by values measured at five random positions in the microhole, and a value of a relative AIF was calculated by dividing the AIF values measured at each condition by the value in the reference condition.

## 3. Results and Discussion

**Figure 1:**
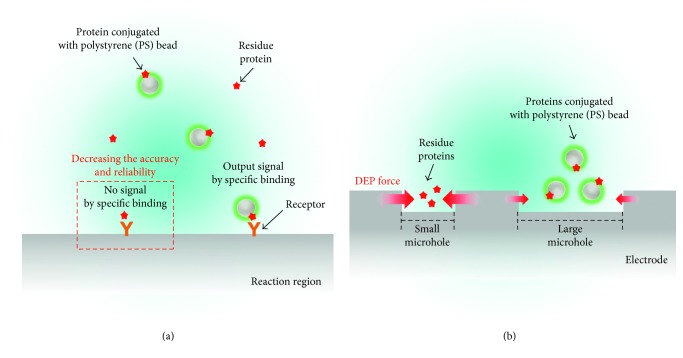
Schematic illustration of a simple separation method of the protein and protein conjugated with polystyrene (PS) beads. (a) Intensity of fluorescence by a specific binding of the protein conjugated with PS beads decreased due to a specific binding of the nonconjugated protein, expressed as residue protein. (b) Residue protein and protein conjugated with PS beads were separated by the dielectrophoresis (DEP) force in different microholes, respectively.

Protein, nonconjugated with PS beads after labeling, does not emit the fluorescence but binds to the receptor specifically so that it impedes the specific binding between the ideally conjugated protein with PS beads and receptor, followed by decreasing the accuracy and reliability in the molecules' analyzing and tracking process (Figure 1(a)). Thus, the nonconjugated protein, namely, the residue protein, should be separated from the protein conjugated with PS beads. When alternating current (AC) voltage is applied to the electrode with microholes, the protein is allocated into each microhole according to the intensity of the DEP force that occurred in each microhole. The intensity of the DEP force is related to the diameter of the molecules and the distance between the electrodes, namely, the size of microholes, as described in (4), and consequently allocates different molecules into each microhole, respectively: residue protein, smaller than the PS beads, is allocated into the small microhole, whereas the PS beads are placed in the large microhole—two molecules separate into small and large microholes, respectively (Figure 1(b)).

**Figure 2:**
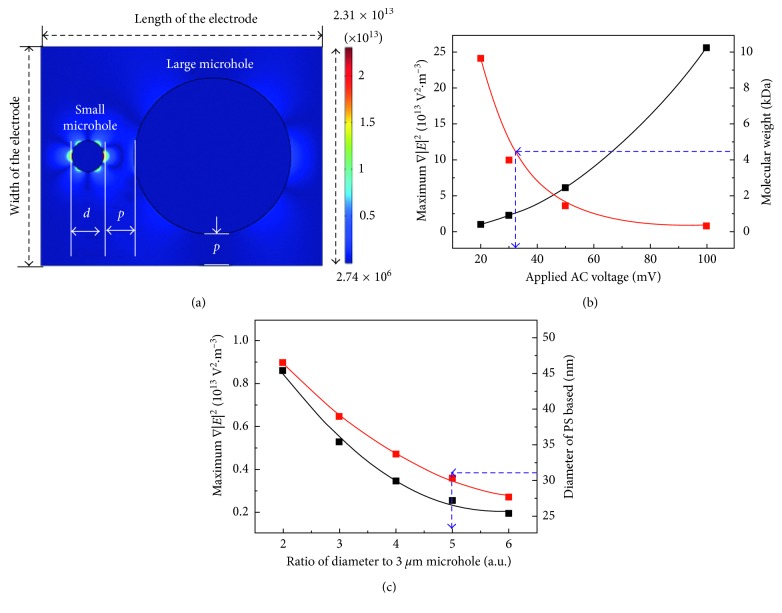
Simulation for separating the molecules by DEP force in the two microholes. (a) Distribution of ∇|*E*|^2^ at the top view of the electrode. The diameter of the small microhole and pitch between two microholes were represented as “*d*” and “*p*,” respectively. (b) According to the applied voltage, maximum intensity of ∇|*E*|^2^ occurring in the 3 *μ*m hole increased, whereas molecular weight of the protein, allocated into the microhole, decreased. (c) Maximum intensity of ∇|*E*|^2^ in the other microhole and the diameter of PS beads, allocated into the hole, decreased according to the increase of the diameter.

In order to separate the residue protein and conjugated protein with PS beads in each microhole, 4.5 kDa of amyloid beta, whose diameter was calculated to be approximately 2 nm, was used as a residue protein, and the conjugated protein with PS beads was simplified to just PS beads. The length and width of the electrode were fixed to 27 *μ*m and 21 *μ*m, respectively, and the diameter of the small microhole, *d*, and pitch between two microholes, *p*, were fixed to 3 *μ*m. The intensity of the applied AC voltage and size of microholes were optimized via the COMSOL simulation (Figure 2(a)). Firstly, maximum intensity of ∇|*E*|^2^ in the small microhole was simulated according to the applied AC voltage (Figure 2(b)). Black line and scatter showed the maximum intensity of ∇|*E*|^2^ that occurred in the small microhole, and red line and scatter indicated the size of protein, which was allocated into the small microhole, depending on the intensity of the applied AC voltage. The intensity of ∇|*E*|^2^ increased parabolically and size of the protein decreased accordingly. The results signified that approximately 30 mV voltage, which resulted in ∇|*E*|^2^ with intensity approximately 2.310 × 10^13^ V^2^·m^−3^, was required to place 2 nm of the protein in the 3 *μ*m hole. Also, the maximum intensity of ∇|*E*|^2^ that occurred in the other microhole was simulated according to the size of the other microhole at the condition that applied 30 mV AC voltage (Figure 2(c)). The diameter of the other microhole was expressed as a ratio to the diameter for the 3 *μ*m hole, and the intensity of ∇|*E*|^2^ decreased according to the increase in the ratio of the diameter. In the 6 *μ*m microhole (the ratio was 2), intensity of ∇|*E*|^2^ was about 0.861 × 10^13^ V^2^·m^−3^ and it is too strong to allocate 30 nm of the PS beads into the hole. ∇|*E*|^2^ was approximately 0.528 × 10^13^ V^2^·m^−3^, 0.347 × 10^13^ V^2^·m^−3^, 0.255 × 10^13^ V^2^·m^−3^, and 0.194 × 10^13^ V^2^·m^−3^ in each value of the ratio, and an optimized size of the microhole for placing 30 nm of the PS beads into the hole was verified to be 15 *μ*m. Thus, the two microholes for separating the residue protein and protein conjugated with PS beads were optimized to 3 *μ*m and 15 *μ*m, respectively, whose difference in the intensity was approximately 9.059-fold.

**Figure 3:**
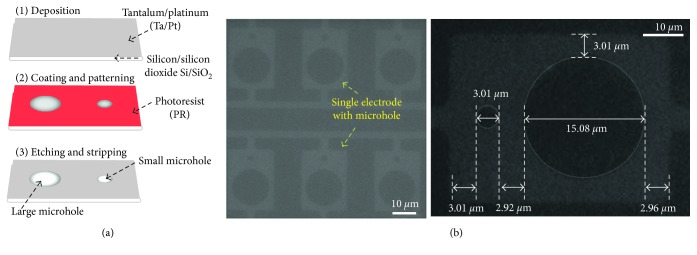
Fabrication of the single electrode consisting of two different-sized microholes by MEMS technology. (a) Schematic illustration of the fabrication process of the microholes. (b) Microscopic image of the two different microholes.

Two microholes in the electrode were produced via a standard MEMS process on a 4-inch silicon (Si) wafer. The fabrication process consisted of 3 steps (Figure 3(a)), and details of the process are described in Material and Methods. The diameter of the fabricated small and large microholes was approximately 3 *μ*m and 15 *μ*m, respectively, and pitch between two microholes was about 3 *μ*m (Figure 3(b)).

**Figure 4:**
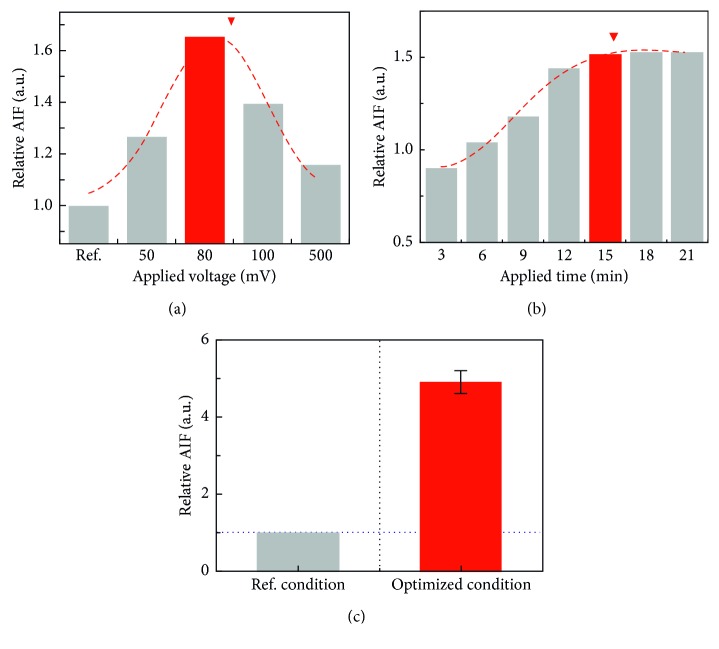
Optimization of the DEP condition by measuring the average intensity of fluorescence (AIF) of the PS beads in the 15 *μ*m hole. Relative AIF was verified according to (a) the applied voltage and (b) the applied time of AC voltage. (c) Relative AIF by the PS beads in the 15 *μ*m hole was compared in each reference and optimized DEP condition.

Various conditions of DEP, intensity of the DEP force and applied time, were optimized by measuring the relative AIF resulting from placing the PS beads in the 15 *μ*m hole. In order to optimize the intensity of the DEP force, the applied frequency required for the DEP force to occur was fixed at 50 MHz. Firstly, relative AIF was measured in various applying voltage conditions ranging from 0 V (ref.) to 500 mV, and consequently, it was maximized at 80 mV (Figure 4(a)). The values were approximately 1, 1.265, 1.655, 1.396, and 1.1604 in ref., 50 mV, 80 mV, 100 mV, and 500 mV, respectively. The results indicated that the PS beads were most effectively placed in the microhole by the DEP force induced by the applied voltage 80 mV; consequently, the intensity of the applied voltage was settled to 80 mV. Also, in order to optimize the applied time condition of DEP force, the relative AIF was measured according to time every 3 minutes up to 21 minutes (Figure 4(b)). The AIF increased gradually depending on the time up to 15 min and was saturated afterward: each value of the AIF was approximately 0.907, 1.048, 1.184, 1.450, 1.526, 1.535, and 1.536 according to the applied time. Hence, the applied voltage and time were optimized to 80 mV and 15 minutes, respectively, and the relative AIF was observed to be approximately 4.908 ± 0.299 in the optimized condition (Figure 4(c)). The result demonstrated that the PS beads were allocated into the 15 *μ*m hole effectively.

**Figure 5:**
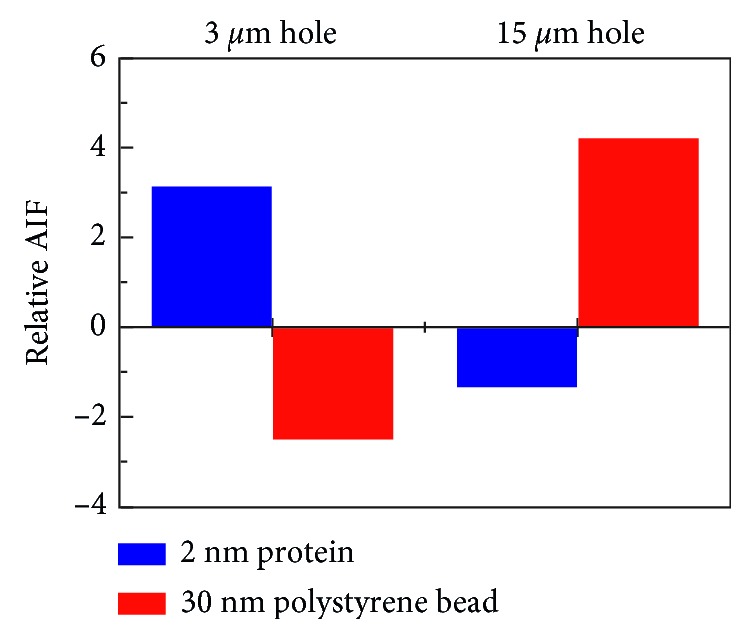
Relative AIF by 2 nm of the protein and 30 nm of the PS beads in 3 *μ*m and 15 *μ*m holes, respectively.

Finally, based on these results, a separation of the protein and PS beads in 3 *μ*m and 15 *μ*m holes, respectively, was demonstrated (Figure 5). It was also confirmed by comparing the AIF by each molecule in the two microholes at the previous optimized condition. The relative AIF by 2 nm of the protein in the 3 *μ*m hole was a positive value, but the value by 30 nm of the PS beads was negative, and the values were approximately 3.143 and −1.346, respectively, whereas in the 15 *μ*m hole, the relative AIFs by the protein and the PS beads showed an opposite sign compared with the previous values, and the values were approximately −2.515 and 4.211, respectively. The negative value of the AIF indicated that the molecules were moving far away owing to the strong DEP force in the microhole, and the positive value of the AIF signified that the molecules were attracted and trapped into the microhole by the DEP force. Thus, the results signified that the DEP force allocated 2 nm of the protein and 30 nm of the PS beads into 3 *μ*m and 15 *μ*m holes, respectively. The results demonstrated that 2 nm of the protein and 30 nm of the PS beads were separated by DEP force in each microhole, effectively.

## 4. Conclusions

In this paper, a simple method for separation between 2 nm of the protein and PS beads into different microholes, respectively, by the DEP force was demonstrated. In order to separate two molecules, the diameter of the two microholes was simulated and the intensity of the DEP force induced in the microholes was calculated via simulation. The optimized diameter of the two microholes was 3 *μ*m and 15 *μ*m, and a difference in the DEP force between two microholes was approximately 9.059-fold. The condition of the DEP force to separate two molecules was optimized experimentally: intensity of the AC voltage was 80 mV and the applied time was 15 minutes. The molecules which were separated by the DEP force in each microhole were verified by measuring the relative AIF by each molecule. In 3 *μ*m and 15 *μ*m holes, the AIFs were approximately 3.143 and −1.346 by 2 nm of the protein and about −2.515 and 4.211 by 30 nm of the PS beads, respectively. Consequently, the results demonstrated that 2 nm of the protein and 30 nm of the PS beads were separated by DEP force in each microhole, effectively. Our method has high expandability in separation of various-sized molecules, and furthermore, it is applicable for verification of the labeling efficiency.

## Data Availability

The data used to support the findings of this study are available from the corresponding author upon request.
